# Food insecurity among Latina women in Idaho and Washington: structural vulnerability, psychosocial factors, and cardiometabolic risk

**DOI:** 10.3389/fnut.2026.1945643

**Published:** 2026-09-23

**Authors:** Rita Franco, Evelyn Roldán Vernon, Brittany Rushing, Kevin Bui, Patricia Villamediana, Adamarie Márquez Acevedo, Ginny Lane

**Affiliations:** 1 Margaret Ritchie School of Family and Consumer Sciences, University of Idaho, Moscow, ID, United States; 2 University of Washington School of Medicine, Seattle, WA, United States; 3 Department of Mathematics and Statistical Science, University of Idaho, Moscow ID, United States; 4 National Institutes of Health (NIH) Center of Biomedical Research Excellence (COBRE) in Nutrition and Women’s Health, University of Idaho, Moscow, ID, United States; 5 Department of Animal, Veterinary and Food Sciences, University of Idaho, Moscow, ID, United States

**Keywords:** adverse childhood experiences, agricultural workers, food insecurity, health equity, Latina women, obesity, prediabetes, social support

## Abstract

**Introduction:**

Latina women in the United States face overlapping nutritional, social, and cardiometabolic vulnerability, yet how food insecurity, excess weight, and cardiometabolic risk co-occur in underrepresented communities remains insufficiently characterized. We examined food insecurity among Latina women in Idaho and Washington as a marker of vulnerability and situated it within a broader context of excess weight, prediabetes risk, and structural and psychosocial disadvantage.

**Methods:**

Cross-sectional survey data were collected from 199 self-identified Latina women recruited through a stratified, address-based sampling design together with community outreach. Food insecurity was assessed using the Hunger Vital Sign; the cardiometabolic profile by body mass index, overweight or obesity, and the Bang prediabetes risk score; and dietary quality using the Mini-EAT. Structural and psychosocial factors included farmworker household status, education, age, social support, discrimination, depressive symptoms, and adverse childhood experiences, with an exploratory analysis of acculturative stress among immigrant participants. Data analysis included modified Poisson regression (prevalence ratios, PR) and proportional-odds ordinal regression.

**Results:**

Food insecurity was common (43.3%), and overweight or obesity was highly prevalent (78.9%). In the final model, food insecurity was more prevalent among women in farmworker households (PR 1.65, 95% CI 1.07–2.55) and those with less than a high-school education, though this estimate was based on few women (PR 1.87, 95% CI 1.10–3.19), and less prevalent among women with higher social support (PR 0.99 per point, 95% CI 0.98–1.00). A higher burden of adverse childhood experiences was associated with greater food-insecurity severity (cumulative OR 1.18, 95% CI 1.01–1.38). Among immigrant women, acculturative stress was associated with food insecurity (PR 1.61, 95% CI 1.28–2.03). Dietary quality was broadly suboptimal, and its association with food-insecurity status was modest and did not establish directionality, and food insecurity was not clearly associated with prediabetes risk.

**Discussion:**

These findings illustrate the food insecurity–obesity paradox in an understudied regional population, in which a high prevalence of food insecurity coexists with a high prevalence of overweight and obesity.

## 1 Introduction

Food insecurity and obesity frequently coexist within the same households, a pattern observed across many populations and especially among women (1, 2). Food insecurity, structural disadvantage, and cardiometabolic risk have been documented among Latina and farmworker populations in the United States (3–5), but how they cluster among Latina women in the rural and urban communities of the Pacific Northwest remains unclear. The present study seeks to fill this research gap.

Food security exists when all people, at all times, have physical, social, and economic access to sufficient, safe, and nutritious food that meets their dietary needs and food preferences for an active and healthy life (6). International human-rights guidance further specifies that adequate food must be acceptable within a given culture, taking into account social meanings and non-nutritional values that people attach to the food they eat (7). Food insecurity has been associated with obesity, particularly among women (1, 2), and with type 2 diabetes risk among Latinas (3). Among Latinas at risk for diabetes, food insecurity is also linked to psychosocial distress, poor sleep, and cyclical patterns of food access that may shape diabetes-related risk (8, 9). Limited budgets can push households toward relying on inexpensive, energy-dense foods of lower nutritional quality (1, 2), while the stress of going without food and the coping strategies required add a psychosocial dimension (10). Among Latina populations, food insecurity has also been tied to poor cardiometabolic health (11, 12). This pattern points to an apparent paradox, in which food insecurity coexists with overweight and obesity rather than underweight from possible nutritional deficits (1, 2). That paradox shows how deprivation and excess can coexist in the same households and the same bodies.

Latina women in the United States carry an elevated and overlapping burden of food insecurity, overweight and obesity, and type 2 diabetes (3, 13). In the United States diagnosed diabetes is far more common among Hispanic adults than among non-Hispanic White adults (11.7% versus 6.9%, age-adjusted) (14). Several structural conditions shape this burden. For many Latina families connected to agricultural work, low and seasonal earnings, hazardous working conditions, and limited access to food and health care can compound vulnerability (4, 15). Food insecurity in farmworker households was documented in the early 2000s (15–17) and remains common among rural Latino immigrant families (5), coexisting with a heavy burden of obesity and other diet-related chronic conditions (4). In addition, immigration-related stress, discrimination, and lower educational and economic resources compound these vulnerabilities across Latino populations (18–20). Persistent diabetes disparities by ethnicity and rurality are recognized (21). Still, existing literature does not explain how food insecurity, structural vulnerability, psychosocial adversity, and cardiometabolic risk cluster among Latina women in the rural and urban communities of the Pacific Northwest. The regional context may also shape these vulnerabilities. In Idaho, much of the Hispanic population is concentrated in agricultural areas along the Snake River Plain (22), and research among Latina farmworkers has identified food provisioning, childcare responsibilities, and social isolation as challenges to wellbeing (23). Public health professionals working with Hispanic communities have also described geographic differences in barriers to service use. In Idaho, these included cultural insensitivity, limited awareness of available services, and challenges building trust in communities with fewer Hispanic residents (24). Recent evidence from Washington similarly documents difficulty affording sufficient food among farmworkers (25). Few studies have examined structural and psychosocial factors associated with food insecurity among Latina women in Idaho and Washington. By examining these factors together, this study provides context-specific evidence from an understudied regional population and complements findings from national studies.

A substantial body of literature links food insecurity to diabetes and related conditions in Latino populations along with the underlying structural and syndemic forces (3, 11, 20, 26). However, much of the existing literature examines individual social needs, exposures, or biomedical risk factors, rather than considering food insecurity together with structural vulnerability, psychosocial adversity, and cardiometabolic risk in the same study (26, 27). This study treats food insecurity not only as a risk factor for disease, but as a marker of structural and psychosocial vulnerability, coexisting with excess weight and cardiometabolic risk among Latina women across rural and urban communities in Idaho and Washington. Syndemic frameworks describe how co-occurring social adversities may cluster and interact to shape health rather than acting as independent risks (20, 28). We draw on this perspective to motivate the study rather than to test it: our analyses examine associations of food insecurity with structural and psychosocial factors while characterizing coexisting excess weight and cardiometabolic risk, without evaluating statistical interaction or mediation among these factors. Within this approach, food insecurity represents deeper structural and psychosocial vulnerability, while cardiometabolic risk represents expression of that inequality. This perspective shifts the analytic question from whether single factors raise risk toward how disadvantage accumulates in the same women within the same communities.

Guided by this perspective, the study pursued three aims. First, we identified the structural and psychosocial factors strongly associated with food insecurity among Latina women in Idaho and Washington. Second, we described how food insecurity coexists with excess weight and prediabetes-related risk. Third, in an exploratory analysis restricted to immigrant participants, we examined the association between acculturative stress and food insecurity. Dietary quality, assessed with the Mini-EAT, was analyzed as a complementary nutritional outcome.

## 2 Materials and methods

### 2.1 Study design and setting

We conducted a cross-sectional analysis of survey data collected from Latina women in Idaho and Washington. The survey focused on type 2 diabetes risk and the social, environmental, psychosocial, and overall health status in women with and without diabetes.

### 2.2 Formative community consultations

Our team held community consultations to ensure the survey questions aligned with the priorities and lived experiences of Latina women. The consultations took place in Fall 2024 in Jerome and Nampa, Idaho, and included about 20 participants in total, consisting of Latina women, healthcare providers, and community service providers. The community consultations confirmed the relevance of the structural and psychosocial domains in the survey and informed decisions about content, wording, and participant recruitment.

### 2.3 Recruitment and sampling

We recruited self-identified Latina or Hispanic women, 18 years or older, living in Idaho or Washington. Recruitment combined four outreach strategies: mailed invitations sent through an address-based sampling design described further below, promotion by community partners, social media posts, and in person at community health fairs, including events in Othello, WA (19 September 2025) and Royal City, WA (10 October 2025). The online survey was available in English and Spanish, with responses collected between September and December 2025.

The mailed invitations followed a formal address-based sampling design. Using the Marketing Systems Group GENESYS frame, census tracts were selected within state and rural strata by probability proportional to size, and addresses were sampled by Zip+4 within each tract. Marketing Systems Group provided a listed sample of 4,029 households with a likely Latina resident across the two states, and each household received a mailed invitation letter with a QR code and link to the survey.

Each recruitment strategy used a separate survey link. We removed responses that were flagged as automated submissions (a security alert), lacked consent, did not identify the respondent as female or as Latina/Hispanic, were empty, were completed in under 10 min, or had a ZIP code inconsistent with the eligibility area. Due to security concerns and unvalidated addresses from the open-link social media posts, surveys entered using this methodology were not retained in the final sample. Ineligible participant responses were removed on an ongoing basis during data collection when they were flagged as ineligible. The number removed under each criterion cannot be determined retrospectively because a cumulative log was not kept. Response rates cannot be computed for the open-link strategies. The final analytic sample comprised 199 surveys, predominantly from the address-based mailed distribution group.

To characterize the rural and urban composition of the sample, each participant’s ZIP code was mapped to their county of residence and classified using the 2023 USDA Economic Research Service Rural-Urban Continuum Codes (29). Counties coded 4 through 9 were considered non-metropolitan (rural), and counties coded 1 through 3 were considered metropolitan (urban).

### 2.4 Measures

Participants could skip any question other than the qualifying criteria (sex, Latina, and consent questions), so the number of women with complete data varied across measures.

#### 2.4.1 Primary outcome: food insecurity

Food insecurity was measured with the Hunger Vital Sign, a two-item screener adapted from the U.S. Household Food Security Survey Module (30). Women who answered “often true” or “sometimes true” to either item were classified as food insecure. A three-level severity variable (food secure, marginal, food insecure) was derived for a sensitivity analysis.

#### 2.4.2 Cardiometabolic measures

Body mass index (BMI) was calculated from self-reported height and weight, and women with a BMI of 25 kg/m^2^ or higher were classified as having overweight or obesity. Prediabetes risk was estimated from the Bang score (31), a validated clinical risk score for type 2 diabetes.

#### 2.4.3 Structural vulnerability

Structural and social conditions were assessed with items from the PRAPARE tool (Protocol for Responding to and Assessing Patients’ Assets, Risks, and Experiences), specifically farmworker household status, educational attainment (less than high school, high school or equivalent, some college or more), household income (low income defined as less than $25,000), and survey language.

#### 2.4.4 Psychosocial vulnerability

Social support was measured with the Medical Outcomes Study (MOS) Social Support Survey, scored as a 0–100 index, with higher scores indicating greater perceived support (32). Discrimination was measured with the Everyday Discrimination Scale (33). Depressive, anxiety, and stress symptoms were measured with the Depression Anxiety Stress Scales-21 (DASS-21), with subscale scores doubled per standard scoring so that each ranges from 0 to 42 (34). Adverse childhood experiences (ACE) were measured with the 10-item ACE questionnaire, with a high burden defined as four or more (35).

#### 2.4.5 Acculturative stress (immigrant subgroup)

Among women who identified as immigrants, acculturative stress was measured with the Stress of Immigration Survey (36), scored as the mean of 21 items. Acculturative stress was modeled as a continuous score and treated as exploratory because analyses were restricted to the small immigrant subgroup.

#### 2.4.6 Dietary quality

Diet quality was measured with the Mini-EAT food-group frequency (37). Scores were computed with the Mini-EAT linear algorithm and coefficients obtained from the instrument authors, and classified as healthy, intermediate, or unhealthy following the instrument’s validated interpretation, in which higher scores indicate a healthier diet (37).

#### 2.4.7 Additional measures

The survey also included measures of unmet social needs (PRAPARE) (38), intimate partner violence (HITS) (39), and physical activity (GPAQ) (40). These were used descriptively or as sensitivity covariates rather than as primary exposures.

### 2.5 Instrument translation and administration

The survey was available online in English and Spanish so participants could complete it in either language. Two bilingual team members independently translated the survey into Spanish, and a group of university students from several Latin American countries reviewed the Spanish version for clarity and regional appropriateness before the survey was launched. Validated questionnaires were used with permission or under open license.

### 2.6 Statistical analysis

Food insecurity was the primary outcome. We estimated associations with structural and psychosocial factors using modified Poisson regression with robust standard errors to yield prevalence ratios (41). We fit separate structural and psychosocial models and a final parsimonious model that combined a pre-specified set of structural and psychosocial predictors. Because the three DASS-21 subscales were strongly intercorrelated, we fitted three parallel psychosocial models, each including discrimination, social support, ACE count, low income, age, and one DASS-21 subscale (depression, anxiety, or stress). Prevalence ratios are reported with 95% confidence intervals, and continuous predictors are expressed per one-unit increase.

All models used unweighted complete-case analysis, with participants excluded from a given model if they were missing data on any variable included in that model. Thus, sample sizes varied across analyses. We modeled the three-level food-insecurity severity variable with ordinal proportional-odds regression, reporting cumulative odds ratios, and examined the proportional-odds assumption. Among immigrant women, we examined acculturative stress in a separate modified Poisson model; this analysis was exploratory because of the smaller subgroup.

Prediabetes risk, measured with the Bang score, was analyzed as secondary context. We fit modified Poisson models for high Bang risk and robust linear models for the continuous Bang score. These analyses were pre-specified as contextual and were expected to be imprecise, given the smaller number of women with complete Bang data. Physical inactivity, a component of the Bang score, was examined descriptively rather than as an independent predictor. Dietary quality (Mini-EAT) was examined in two ways: as a predictor added to the parsimonious food-insecurity model, and as a continuous outcome in robust linear regression models, comprising a structural model and a parsimonious model that also included psychosocial correlates. These two parameterizations describe the same cross-sectional association and are not independent analyses. As a further exploratory analysis, we examined self-reported clinician-diagnosed diabetes (includes type 1 and type 2) using Firth penalized logistic regression adjusted for age, given the small number of diabetes cases.

An *a priori* sample-size calculation was conducted for the parent study using G*Power (42) for the originally planned linear multiple-regression analyses, assuming a medium effect size (f^2^ = 0.15), α = 0.05, and 95% power. The parent protocol specified an analytic sample of 310 women, with equal recruitment by diabetes status, and a recruitment target of 342 to allow for attrition. The present analysis differs from the design underlying that calculation, using modified Poisson regression with food insecurity as the primary outcome. The final analytic sample comprised 199 participants, and the final parsimonious model included 124 participants with 48 food-insecurity events. Accordingly, estimates are interpreted with emphasis on effect magnitude and precision, including 95% confidence intervals. We confirmed no difference between participants included and excluded in the final model by comparing participants included in (*n* = 124) and excluded from (*n* = 75) the final model using standardized mean differences, and repeated the final model after multiple imputation by chained equations (*m* = 30) as a sensitivity analysis. Stability of the final parsimonious model was assessed with a non-parametric bootstrap (5,000 resamples), comparing bootstrap percentile intervals with the robust Wald intervals.

Primary analyses used complete cases without imputation; multiple imputation was applied only as a sensitivity analysis. The acculturative-stress items were administered only to immigrant women by design, and other measures had some item non-response; analyzable sample sizes therefore varied across models. We assessed model adequacy through convergence, dispersion, variance inflation factors, and Cook’s distance. Analyses were conducted in R, with a two-sided significance level of 0.05. We emphasized the size and precision of associations rather than significance thresholds alone.

### 2.7 Ethics

The study was reviewed and approved by the University of Idaho Institutional Review Board (Protocol 25-173). Participation was voluntary, and all participants provided informed consent before beginning the survey.

## 3 Results

### 3.1 Sample characteristics

The final sample included 199 Latina women. All participants self-identified as Hispanic or Latina, consistent with the eligibility criterion; about one in four (48 of 199) also identified as White, and most did not report a separate racial category. Fifty-seven of the 196 women who answered the immigration item (29.1%) identified as immigrants. Twenty of 199 (10.1%) completed the survey in Spanish, while 47 of the 192 women who reported a language preference (24.5%) indicated they were most comfortable speaking Spanish. Food security data were available for 187, of whom 81 (43.3%) were classified as food insecure. Overweight or obesity was common, affecting 127 of 161 women with an available body mass index (78.9%). The sample spanned both states and a mix of rural and urban communities: of the 193 women with a valid ZIP code, 87 (45%) lived in non-metropolitan counties.

Food insecurity was linked with greater structural and psychosocial disadvantage (Table 1). Food-insecure women were younger than food-secure women (median age 32 versus 35 years) and more often lived in farmworker households (36% of food-insecure versus 14% of food-secure women). They had lower educational attainment, with 16% reporting less than a high-school education compared with 6.6% of food-secure women, and their household income was concentrated in the lower income categories. Psychosocial differences were pronounced: food-insecure women reported higher discrimination scores (median 25 versus 18), lower social support (median MOS score 57 versus 87), higher depressive, anxiety, and stress symptoms, and a higher burden of adverse childhood experiences (median ACE count 3.5 versus 1.0).

**TABLE 1:** Participant characteristics by food-security status (*N* = 187).

Characteristic	Food secure (*N* = 106)	Food insecure (*N* = 81)	*P*
Age, years	35.0 (28.0–49.0)	32.0 (26.0–39.0)	0.016
Survey completed in Spanish	6/106 (5.7)	10/81 (12)	0.12
Farmworker household	15/106 (14)	29/81 (36)	<0.001
Education			0.017
Some college or more	70/106 (66)	41/81 (51)	–
High school/GED	29/106 (27)	27/81 (33)	–
Less than high school	7/106 (6.6)	13/81 (16)	–
Household income			0.010
<$25,000	14/94 (15)	15/76 (20)	–
$25,000–49,999	23/94 (24)	33/76 (43)	–
$50,000–99,999	38/94 (40)	20/76 (26)	–
≥$100,000	19/94 (20)	8/76 (11)	–
Everyday Discrimination score	18.0 (14.0–25.0)	25.0 (17.0–31.0)	<0.001
Social support (MOS) score	86.8 (65.8–97.4)	56.6 (40.8–84.2)	<0.001
DASS-21 depression	6.0 (2.0–12.0)	12.0 (5.0–21.0)	<0.001
DASS-21 anxiety	6.0 (2.0–12.0)	12.0 (4.0–20.0)	<0.001
DASS-21 stress	12.0 (6.0–16.0)	16.0 (8.0–26.0)	0.002
ACE count	1.0 (0.0–4.0)	3.5 (2.0–7.0)	<0.001
Physical inactivity	36/99 (36)	32/71 (45)	0.3
Body mass index, kg/m^2^	29.1 (24.6–33.2)	29.1 (25.8–35.4)	0.2
Overweight or obesity (BMI ≥ 25)	67/91 (74)	56/66 (85)	0.12
High prediabetes (Bang) risk	22/83 (27)	10/57 (18)	0.2

Continuous variables are median (Q1–Q3); categorical variables are *n*/*N* (%). Denominators vary because of item missingness; 12 women with missing food-security status are excluded. *P*-values from exact Wilcoxon–Mann–Whitney tests (continuous variables), exact linear-by-linear trend tests (ordinal variables: education and household income), and Fisher’s exact tests (binary and nominal variables). MOS, Medical Outcomes Study; DASS-21, Depression Anxiety Stress Scales-21; ACE, adverse childhood experiences.

Excess weight was common regardless of food-security status. Overweight or obesity affected 85% of food-insecure women and 74% of food-secure women, and median body mass index was similar in the two groups (29.1 kg/m^2^). High prediabetes risk based on the Bang score was more common among food-secure than food-insecure women (27% versus 18%), although the difference was not statistically significant (*p* = 0.2; Table 1).

### 3.2 Structural correlates of food insecurity

In the adjusted structural model (Table 2), food insecurity was more prevalent among women in farmworker households (PR 1.53, 95% CI 1.08–2.18) and among women with less than a high-school education compared with some college or higher (PR 1.87, 95% CI 1.19–2.93). Each additional year of age was associated with lower food-insecurity prevalence in this model (PR 0.97, 95% CI 0.95–0.99), although this association attenuated after full adjustment in the parsimonious model (PR 0.98, 95% CI 0.97–1.00). The binary low-income indicator was not clearly associated with food insecurity after adjustment (PR 0.89, 95% CI 0.58–1.36), although the broader income distribution shifted toward lower categories among food-insecure women. Survey language was not associated with food insecurity (PR 1.17, 95% CI 0.68–2.01).

**TABLE 2:** Structural and psychosocial correlates of food insecurity: crude and adjusted modified Poisson models.

Predictor	Crude	Structural adjusted *n* = 156, 70 events	Psychosocial adjusted (depression) *n* = 113, 45 events	Final parsimonious *n* = 124, 48 events
Structural				
Farmworker household	1.81 (1.34–2.46) *p* < 0.001	1.53 (1.08–2.18) *p* = 0.017	—	1.65 (1.07–2.55) *p* = 0.023
Education: less than high school^a^	1.76 (1.18–2.63) *p* = 0.006	1.87 (1.19–2.93) *p* = 0.007	—	1.87 (1.10–3.19) *p* = 0.021
Education: high school/GED^a^	1.31 (0.91–1.88) *p* = 0.152	1.18 (0.81–1.70) *p* = 0.391	—	1.29 (0.82–2.04) *p* = 0.272
Low income (<$25,000)	1.20 (0.80–1.78) *p* = 0.380	0.89 (0.58–1.36) *p* = 0.588	0.93 (0.50–1.72) *p* = 0.811	—
Survey completed in Spanish	—	1.17 (0.68–2.01) *p* = 0.581	—	—
Psychosocial				
Everyday Discrimination score (per point)	1.04 (1.02–1.05) *p* < 0.001	—	1.00 (0.97–1.03) *p* = 0.866	1.00 (0.97–1.03) *p* = 0.920
Social support, MOS score (per point)	0.99 (0.98–0.99) *p* < 0.001	—	0.99 (0.98–1.00) *p* = 0.037	0.99 (0.98–1.00) *p* = 0.028
ACE count (per point)	1.14 (1.07–1.21) *p* < 0.001	—	1.06 (0.98–1.15) *p* = 0.137	1.05 (0.97–1.14) *p* = 0.216
DASS-21 depression^b^ (per point)	1.03 (1.02–1.04) *p* < 0.001	—	1.01 (0.99–1.03) *p* = 0.485	1.02 (1.00–1.04) *p* = 0.091
DASS-21 anxiety^b^ (per point)	1.03 (1.02–1.05) *p* < 0.001	—	—	—
DASS-21 stress^b^ (per point)	1.03 (1.01–1.04) *p* < 0.001	—	—	—
Shared covariate				
Age (per year)	—	0.97 (0.95–0.99) *p* < 0.001	0.98 (0.96–1.00) *p* = 0.043	0.98 (0.97–1.00) *p* = 0.067

PR, prevalence ratio; CI, confidence interval; MOS, Medical Outcomes Study; DASS-21, Depression Anxiety Stress Scales-21; ACE, adverse childhood experiences. All models are modified Poisson regressions with robust variance. Crude models were fitted one predictor at a time, with sample sizes ranging from 134 to 187 depending on item non-response; crude models were not fitted for age or survey language. Em dashes indicate that a predictor was not included in that model. Reference categories: not a farmworker household, some college or more, income ≥ $25,000, survey in English.

^a^Versus some college or more.

^b^Depression, anxiety, and stress were entered in three parallel psychosocial models rather than simultaneously, because the subscales are strongly intercorrelated. The depression model is shown here because depression is the subscale carried into the final parsimonious model; full coefficients for all three parallel models appear in Supplementary Table 4.

### 3.3 Psychosocial correlates of food insecurity

In unadjusted models (Table 2), higher discrimination, lower social support, higher depressive, anxiety, and stress symptoms, and a higher burden of adverse childhood experiences were each associated with food insecurity. After adjustment, social support was the most consistent correlate (Table 2): in the final parsimonious model, each additional point on the social support score was associated with lower food-insecurity prevalence (PR 0.99, 95% CI 0.98–1.00), which corresponds to roughly 10% lower prevalence for every 10-point higher score. Adverse childhood experiences showed a weaker association that remained above one in the parsimonious model (PR 1.05, 95% CI 0.97–1.14) and reached significance for food-insecurity severity in the ordinal model (cumulative OR 1.18, 95% CI 1.01–1.38, *p* = 0.042; Supplementary Table 1). Discrimination and the individual depression, anxiety, and stress subscales separated the groups descriptively but attenuated after adjustment, consistent with shared variance among correlated psychosocial measures. Because the three DASS-21 subscales are strongly intercorrelated, they were entered in three parallel psychosocial models rather than simultaneously (Supplementary Table 4). All 5,000 bootstrap resamples converged, and bootstrap percentile intervals were similar to the robust Wald intervals for all coefficients except less-than-high-school education, whose interval widened to include 1 (bootstrap 95% CI 0.95–3.50), reflecting the small number of women in that stratum (12 of 124); this estimate is therefore interpreted as imprecise (Supplementary Table 7). Participants excluded from the final model were more often food-insecure (52.4% versus 38.7%) and more often Spanish-language respondents (17.3% versus 5.6%; Supplementary Table 6), and prevalence ratios pooled across 30 imputed datasets were within 5% of the complete-case estimates, with unchanged interpretation (farmworker household 1.62, 95% CI 1.19–2.20; less than high school 1.95, 95% CI 1.28–2.97).

### 3.4 Acculturative stress among immigrant women

Among immigrant women, higher acculturative stress was associated with food insecurity in an exploratory model (PR 1.61, 95% CI 1.28–2.03). This estimate was based on 40 of the 57 immigrant participants (20 food-insecure) and is exploratory. By contrast, when immigrant status was added to the adjusted structural model, it was estimated imprecisely (PR 1.06, 95% CI 0.72–1.54).

### 3.5 Food-insecurity severity

In the ordinal severity analysis (Supplementary Table 1), higher social support was associated with lower odds of being in a more severe food-insecurity category (cumulative OR 0.98 per point, 95% CI 0.96–0.99), while a higher burden of adverse childhood experiences was associated with greater severity (cumulative OR 1.18, 95% CI 1.01–1.38). Structural factors pointed in the same direction as the binary models, with wider confidence intervals.

### 3.6 Cardiometabolic risk (secondary)

Prediabetes risk was analyzed as secondary context using the Bang score, evaluated both as a binary high-risk indicator and as a continuous score (Supplementary Table 2). High Bang risk was present in 34 of 144 women (23.6%). Food insecurity was not clearly associated with high Bang risk (PR 0.74, 95% CI 0.38–1.43), and across the adjusted models the estimate ranged from 0.49 to 1.08. The Bang score incorporates age, and food-insecure women were younger, so their lower Bang risk may partly reflect this age component rather than a genuinely lower cardiometabolic risk. Models for the continuous Bang score were similarly inconclusive (for example, β−0.14, 95% CI −0.76 to 0.47). These secondary analyses were based on a smaller sample with fewer high-risk events and are interpreted as imprecise rather than as evidence of no relationship.

In exploratory analyses constrained by the small number of diabetes cases, neither food insecurity nor poor dietary quality was clearly associated with self-reported clinician-diagnosed diabetes (food insecurity OR 1.50, 95% CI 0.52–4.32; poor diet quality OR 2.08, 95% CI 0.78–5.53). With only 18–19 events, these models were underpowered, and the confidence intervals were wide (Supplementary Table 3).

### 3.7 Dietary quality

Dietary quality was suboptimal for most participants. Of the 191 women with Mini-EAT data, 23 (12%) scored in the healthy range, 81 (42%) in the intermediate range, and 87 (46%) in the unhealthy range. With dietary quality as the continuous outcome (Table 3), food insecurity was associated with modestly poorer diet (β−2.22, 95% CI −4.43 to −0.01, *p* = 0.051; parsimonious model: β−2.54, 95% CI −5.02 to −0.06, *p* = 0.047; Supplementary Table 5). Farmworker household status was associated with better dietary quality in the structural model (β 3.56, 95% CI 0.71 to 6.42); and attenuated after adjustment for psychosocial covariates (β 2.54, 95% CI −0.34 to 5.41; Supplementary Table 5). This finding requires further interpretation in light of the high risk of food insecurity among this group. Conversely, when poor dietary quality (unhealthy versus healthy or intermediate) was added as a predictor to the parsimonious food-insecurity model, it was not associated with food insecurity (PR 0.99, 95% CI 0.64–1.53, *p* = 0.979), and estimates for all other predictors were unchanged. These analyses represent alternative parameterizations of the same underlying cross-sectional association.

**TABLE 3:** Dietary quality (Mini-EAT score) as the outcome: robust linear regression (*n* = 155).

Predictor	β (95% CI)	*P*
Food insecure	−2.22 (−4.43 to −0.01)	0.051
Farmworker household	3.56 (0.71 to 6.42)	0.016
Age (per year)	0.10 (0.00 to 0.19)	0.050
Low income (<$25,000)	−2.13 (−5.43 to 1.16)	0.207
Education: high school/GED (vs. some college+)	1.87 (−0.58 to 4.32)	0.137
Education: less than high school (vs. some college+)	0.77 (−3.47 to 5.01)	0.722
Survey completed in Spanish	−0.36 (−4.43 to 3.71)	0.862

β, mean difference in Mini-EAT score (0–90 scale); CI, confidence interval. A higher Mini-EAT score indicates a healthier diet. This model reflects the same cross-sectional association reported above and does not provide independent confirmation.

## 4 Discussion

### 4.1 Principal findings

This study examined food insecurity as a marker of vulnerability to poor health among Latina women in Idaho and Washington. Food insecurity was common (43.3%) and coexisted with a high burden of overweight and obesity (78.9%). It was patterned by structural disadvantage, especially farmworker household status, lower educational attainment, and younger age; and by psychosocial adversity, including lower social support and adverse childhood experiences. Among immigrant women, acculturative stress was associated with food insecurity in an exploratory analysis.

### 4.2 The food insecurity–obesity paradox and the double-burden framework

The coexistence of widespread food insecurity with a high prevalence of overweight and obesity is consistent with the food insecurity–obesity paradox, in which food insecurity accompanies excess weight rather than thinness, particularly among women (1, 2). Limited and unstable food budgets can favor inexpensive, energy-dense foods, while the stress of episodic food shortage adds a psychosocial dimension to this pattern (10). In our sample both conditions were highly prevalent but were not statistically linked (overweight or obesity in 85% of food-insecure versus 74% of food-secure women, *p* = 0.12), so their coexistence did not strictly fit the food insecurity-obesity paradox.

This pattern also speaks to the broader double burden of malnutrition framework, defined as the co-existence of overweight, obesity, or diet-related non-communicable diseases with undernutrition in the same individuals, households, or populations (43), often observed in low- and middle-income countries undergoing nutrition transitions (44, 45). We did not measure undernutrition, micronutrient status, or dietary adequacy, and food insecurity is not itself a component of that definition. Food insecurity has instead been described as a shared pathway contributing to multiple forms of malnutrition, simultaneously promoting dietary inadequacy and excess energy intake: among Mexican women of reproductive age, household food insecurity was associated with the co-occurrence of overweight and anemia (46), and among Black women in Brazil it has been linked to higher odds of both underweight and obesity (47). Separately, the coexistence of food insecurity and obesity has itself been termed a double burden among Latino youth in the United States (48), a construct distinct from the double burden of malnutrition. Whether food insecurity coincides with micronutrient inadequacy (49) among Latina women in this region remains untested. Our data show that food insecurity, excess weight, and cardiometabolic risk co-occur in a population shaped by shared structural conditions, rather than as a set of independent problems (20, 28).

Food insecurity was identified in 43.3% of participants, compared with national estimates of 21.9% among households with a Hispanic person and 13.5% among all U.S. households in 2023 (50). These national estimates derive from the Current Population Survey Food Security Supplement, which classifies household food security using 10 items for households without children and 18 items for households with children, whereas we used the two-item Hunger Vital Sign, a screening tool designed to identify households at risk for food insecurity (30). Because the instruments and classification procedures differ, these estimates are considered contextual benchmarks rather than equivalent measures. High prevalence of overweight or obesity among the Latina women surveyed (78.9%) is consistent with the elevated burden of excess weight documented among U.S. Latinx populations (51). The coexistence of markedly elevated food insecurity with this high burden of excess weight in a high-income setting makes this pattern notable.

### 4.3 Structural vulnerability

The strongest structural factor associated with food insecurity was farmworker household status. Agricultural work in the United States is often characterized by low and seasonal income, hazardous conditions, and limited access to food and healthcare, with food insecurity being widely documented among farmworker households (4, 15–17). In our sample, lower educational attainment was also associated with food insecurity (Table 2). Lower educational attainment may reflect broader socioeconomic disadvantage, including fewer employment and income opportunities. This interpretation is consistent with national evidence showing a strong income gradient in food insecurity (50) and with research linking low educational attainment to food insecurity among Latino adults (52). The burden of food insecurity also fell more heavily on younger women, although this association weakened after full adjustment, a pattern consistent with national evidence showing lower food-insecurity prevalence among older adults (50, 53).

The concentration of food insecurity among younger, reproductive-age women may also have implications for maternal and offspring health through dietary, metabolic, and psychosocial pathways (54). Household food insecurity has been associated with adverse early child development, with maternal depression and anxiety modifying this relationship (55). In addition, evidence from a long-term Guatemalan cohort links nutrition in the first 1,000 days to future adult health and human capital (56). High food insecurity among women of reproductive age suggests that early nutritional disadvantage in this population could carry forward by impacting the cardiometabolic health of the next generation. Prioritizing food security among younger, reproductive-age women could protect not only their own health but also that of their children, reinforcing the case for early, family-centered intervention.

### 4.4 Psychosocial vulnerability

Lower social support was the most stable psychosocial correlate of food insecurity across models, although the association was modest (PR 0.99 per point, 95% CI 0.98–1.00). Social support may buffer the material and emotional strain of food insecurity by providing access to tangible resources, such as food or money, and emotional support that reduces stress. More broadly, social capital and community networks may strengthen food security through food and information sharing (57). In a study of low-income U.S. households, community-level social capital, particularly reciprocity among neighbors, was associated with lower odds of experiencing hunger (58). Social support has also been observed to modify the association between food insecurity and depression among Latinos with uncontrolled type 2 diabetes, with higher support buffering depression risk across levels of food insecurity (10). In our analysis, a higher burden of adverse childhood experiences was associated with greater food-insecurity severity, consistent with a life-course view in which early adversity shapes later vulnerability (35). Among immigrant women, acculturative stress was associated with food insecurity in an exploratory subgroup analysis, consistent with evidence linking migration-related stressors to household food insecurity among Hispanic/Latino families (5, 59). Migration can also reshape the food environment itself; qualitative research with immigrant families describes reduced access to open-air fresh-produce markets, greater reliance on large supermarkets, faster-paced routines, and a pervasive presence of fast and snack foods after resettlement (60). Together, these findings describe a vulnerability that is relational and biographical, not only material.

### 4.5 Cardiometabolic risk and dietary quality as systemic inequality

The relationship between food insecurity and prediabetes risk was imprecise in this cross-sectional sample, with wide confidence intervals. The absence of a clear association does not negate cardiometabolic vulnerability. Food insecurity has been linked to obesity, prediabetes, and type 2 diabetes among Latino adults through dietary, stress, and coping pathways (3, 8, 11, 12). The high burden of excess weight among the Latina women sampled is itself a cardiometabolic risk. In this sample, food insecurity was observed alongside a high burden of excess weight and was associated with lower social support, while a higher burden of adverse childhood experiences was associated with greater food-insecurity severity and, among immigrant women, acculturative stress was associated with food insecurity. Syndemic frameworks conceptualize such clustering of nutritional, psychosocial, and structural vulnerabilities as potentially interacting and mutually reinforcing rather than simply additive (20, 28). Our analyses describe co-occurrence and associations rather than interaction or mediation. Future adequately powered, longitudinal studies should evaluate whether these vulnerabilities interact or operate through shared pathways.

Dietary quality was suboptimal for most women, and its association with food-insecurity status was modest. Food insecurity was associated with a modestly less healthy diet with borderline significance (β−2.22, 95% CI −4.43 to −0.01, *p* = 0.051). Farmworker household status, the strongest structural correlate of food insecurity, was associated with better dietary quality (β 3.56, 95% CI 0.71 to 6.42), which may reflect access to fresh produce through agricultural work. Taken together, the broadly suboptimal diet, the weak association with food-security status, and the unexpected better dietary quality observed among farmworkers suggest dietary quality is shaped by structural and environmental conditions, including the food environment navigated by these women, more than by individual food-security status or circumstance. This interpretation aligns with community members’ statements during our formative consultations, where limited time to cook, food costs, and a food environment dominated by inexpensive processed foods were recurrent themes (unpublished author research). Accordingly, efforts to improve dietary quality may need to address the broader food environment and resource constraints these women navigate, alongside targeted support for food-insecure households. This interpretation should be tested in longitudinal studies incorporating direct measures of the food environment.

### 4.6 Implications for intervention and policy

These findings call for an integrated, culturally sensitive response. Routine food-insecurity screening, such as the Hunger Vital Sign (30), belongs in clinical and community settings that serve Latina women and should connect to referral pathways to support food access. Strengthening social support is a plausible and culturally grounded point of leverage to reduce food insecurity: promotora and community health worker programs have improved diet, physical activity, and diabetes-prevention outcomes in Latino communities (61, 62), and produce-prescription programs, including in rural Idaho and Oregon, have improved food access and cardiometabolic markers (63). The potential impact of these programs depends on how they are delivered. Relationship-based home-visiting models implemented at scale in Latin America, such as Peru’s Cuna Más, suggest that trusted, in-person delivery can shift household practices when implemented with fidelity, although this evidence comes from early-childhood interventions rather than nutrition outcomes (64). These successful programs provide a starting point for program adaptation and evaluation with rural and urban Latinas in the Pacific Northwest.

Study participant characteristics also support concrete design choices. Many women had limited formal schooling, so nutrition programming is likely to be more impactful using visual, practical, and demonstration-based formats like cooking demonstrations, shared meals, and printed materials with simple images and QR codes linking to short recipes. Since farm work was associated with both higher food insecurity and, unexpectedly, better dietary quality, interventions for agricultural-worker households might build on existing produce access rather than assuming a uniform dietary deficit. Structural and policy measures should secure stable food access for agricultural-worker households. Acculturative stress among immigrant families may be an additional target, although this rests on an exploratory subgroup analysis.

### 4.7 Strengths and limitations

This study contributes evidence on an under-studied population of Latina women across rural and urban communities of the Pacific Northwest. Strengths include the use of validated instruments administered in English and Spanish, an address-based sampling design, a transparent log of analytic decisions, and measurement across structural and psychosocial domains. The study was also guided by a community advisory committee comprising representatives of healthcare, public health, education, and Latino-serving community organizations, and by formative community consultations held in rural and urban communities, which informed survey content and increased its relevance to the target population. The survey was administered bilingually, with a Spanish version that was independently translated and reviewed for regional appropriateness by native Spanish speakers from several Latin American countries.

Several limitations should be noted. The cross-sectional survey design only identifies associations rather than causal or temporal relationships. Reverse causation is a particular concern for the diet and diabetes analyses, since behavior change following a diabetes diagnosis is plausible. Some analyses, particularly the prediabetes and exploratory immigrant-only models, relied on reduced subsamples with limited precision. Several models were fitted across multiple outcomes without formal correction for multiple comparisons so we emphasized effect estimates and their precision over statistical significance. Data cleaning prioritized data integrity: after open online links produced responses of unverifiable origin, the analytic sample was restricted to responses from verifiable sources. This decision strengthens confidence in the retained data, but it also means that response rates for the open-link strategies could not be computed and that the overall response was low, so the sample may not represent all Latina women in the region. Given its single-region, address-based design, the study is best interpreted as locally grounded evidence rather than as a source of population-level estimates. Food insecurity was assessed with the two-item Hunger Vital Sign, a screening tool that identifies households at risk rather than classifying household food security status as the full USDA module does, and all measures were self-reported. Although household income was collected in four categories, the models used a single binary threshold (below $25,000), which does not capture the fuller income gradient. The Bang prediabetes score was developed and validated in national survey samples and community-based cohorts of predominantly middle-aged and older U.S. adults, and the Mini-EAT was validated in a clinic-based sample from a preventive cardiology and cardiac rehabilitation setting in Minnesota that was 78.3% White and 93.1% college educated. Their performance among younger Latina women, who differ from these validation populations in age, ethnicity, and educational background, is therefore uncertain. The composition of the sample also limits generalizability. Restriction to complete cases compounded this: women excluded for missing data were more often food-insecure, Spanish-language respondents, lower-income, and from farmworker households (Supplementary Table 6). Although 29.1% of participants were immigrants and 24.5% reported being most comfortable speaking Spanish, only 10.1% completed the survey in Spanish, so the sample is weighted toward women able and willing to respond in English. Latina women with limited English proficiency, more recent arrival, or greater social isolation may be under-represented, and these are among the groups most exposed to the structural conditions examined here. Findings involving farmworker households, and particularly the immigrant-only acculturative stress analysis, should be interpreted with caution. Finally, the originally planned comparison of women with and without diabetes was not feasible, as only 19 participants reported diabetes.

### 4.8 Future directions

Longitudinal studies are needed to clarify how structural conditions, psychosocial stressors, dietary patterns, and food insecurity shape cardiometabolic risk over time. Microlongitudinal designs that capture within-month cycling of food access, developed with Latinas at risk for diabetes (9), are well-suited to this question: the Hunger Vital Sign classifies women at a single point in time and cannot detect the episodic character of food insecurity that may drive metabolic risk. Modeling dietary quality as an outcome of structural and environmental conditions would test whether diet in this population is driven more by environment rather than individual choice. Larger samples would support analyses of diabetes-specific outcomes, and intervention studies could evaluate whether strengthening social support and addressing structural disadvantage reduce food insecurity and associated health consequences.

## 5 Conclusion

Food insecurity among Latina women in Idaho and Washington is not simply a problem of limited food access but a marker of broader structural and psychosocial vulnerability. More than two in five women with food-security data screened positive for food insecurity (43.3%), and overweight or obesity was highly prevalent, a coexistence consistent with the food insecurity–obesity paradox in a population underrepresented in nutrition and public-health research. Food insecurity was more prevalent among women in farmworker households and those with less formal education. Lower social support was the most consistent psychosocial correlate of food insecurity, and a higher burden of adverse childhood experiences was associated with greater food insecurity severity. Among immigrant women, acculturative stress showed a strong exploratory association with food insecurity. These patterns indicate that interventions must move beyond food provision to address the social relationships, occupational conditions, and migration-related stressors that shape nutritional vulnerability. The cardiometabolic findings were less definitive: food insecurity was not clearly associated with prediabetes risk in this cross-sectional sample, and dietary quality was broadly suboptimal regardless of food-security status, consistent with diet being shaped by shared structural conditions rather than individual choice. Longitudinal research is needed to clarify how these factors shape metabolic risk over time. Public-health responses should pair routine food-insecurity screening with referral pathways, culturally and linguistically appropriate nutrition support, and community-based strategies that strengthen social support. For agricultural-worker households and younger women of reproductive age, structural measures that stabilize food access and reduce economic and social stress may carry benefits that extend beyond the individual to the health of the next generation.

## Data Availability

The de-identified data supporting the conclusions of this article will be made available by the corresponding author upon reasonable request, subject to institutional and ethical approvals.
