# Supplementary material for: Food insecurity among Latina women in Idaho and Washington: structural vulnerability, psychosocial factors, and cardiometabolic risk
Source: Front Nutr. 2026 Sep 23;13:1945643. doi: 10.3389/fnut.2026.1945643 (PMC13643915; doi:10.3389/fnut.2026.1945643)
Supplement: Supplementary file 1 [file Table_1.docx]

**Supplementary Materials**

**Supplementary Tables**

**Table S1.** Food-insecurity severity: ordinal model (cumulative odds ratios; n = 124).

| **Predictor** | **cOR (95% CI)** | **p** |
| --- | --- | --- |
| Social support (MOS) score (per point) | 0.98 (0.96–0.99) | 0.009 |
| ACE count (per point) | 1.18 (1.01–1.38) | 0.042 |
| Farmworker household | 1.89 (0.72–4.96) | 0.195 |
| Education: less than high school (vs some college+) | 3.07 (0.79–11.94) | 0.106 |
| Education: high school / GED (vs some college+) | 1.73 (0.68–4.40) | 0.252 |
| Everyday Discrimination score (per point) | 1.00 (0.95–1.07) | 0.886 |
| DASS-21 depression (per point) | 1.05 (0.99–1.10) | 0.076 |
| Age (per year) | 0.97 (0.93–1.01) | 0.096 |

*cOR, cumulative odds ratio (proportional-odds ordinal regression); CI, confidence interval. A cOR below 1 indicates lower odds of more severe food insecurity. The proportional-odds assumption was not violated.*

**Table S2.** Secondary cardiometabolic models: prediabetes (Bang) risk.

| **Predictor** | **Estimate (95% CI)** | **p** |
| --- | --- | --- |
| ***Panel A. High Bang risk, modified Poisson (PR; n = 129, 30 events)*** | | |
| Food insecure | PR 0.74 (0.38–1.43) | 0.371 |
| Low income (< $25,000) | PR 1.24 (0.53–2.91) | 0.621 |
| Farmworker household | PR 0.69 (0.25–1.89) | 0.471 |
| ***Panel B. Continuous Bang score, robust linear regression (β; n = 129)*** | | |
| Food insecure | β −0.14 (−0.76 to 0.47) | 0.650 |
| Low income (< $25,000) | β 0.04 (−1.00 to 1.08) | 0.945 |
| Farmworker household | β −0.51 (−1.50 to 0.48) | 0.317 |

*PR, prevalence ratio; β, mean difference in Bang score; CI, confidence interval. The overall Bang descriptive sample was 144 women with 34 high-risk events; the adjusted models here use complete cases (n = 129, 30 events). These secondary models are imprecise.*

**Table S3.** Exploratory self-reported diabetes models.

| **Outcome** | **Predictor** | **Model** | **n / events** | **OR (95% CI)** | **p** |
| --- | --- | --- | --- | --- | --- |
| Self-reported diabetes | Food insecure | Firth logistic, age-adjusted | 170 / 18 | 1.50 (0.52–4.32) | 0.448 |
| Self-reported diabetes | Poor diet quality | Firth logistic, age-adjusted | 173 / 19 | 2.08 (0.78–5.53) | 0.144 |

*OR, odds ratio; CI, confidence interval. Exploratory only: with 18–19 events these models are severely underpowered and the confidence intervals are very wide. Reported for transparency, not as evidence of association.*

**Table S4. The three parallel adjusted psychosocial models for food insecurity.**

| **Predictor** | **Depression model**  **n = 113, 45 events** | **Anxiety model**  **n = 116, 46 events** | **Stress model**  **n = 116, 46 events** |
| --- | --- | --- | --- |
| Everyday Discrimination score (per point) | 1.00 (0.97–1.03)  p = 0.866 | 1.01 (0.98–1.04)  p = 0.583 | 1.01 (0.98–1.04)  p = 0.423 |
| Social support, MOS score (per point) | 0.99 (0.98–1.00)  p = 0.037 | 0.99 (0.98–1.00)  p = 0.022 | 0.99 (0.98–1.00)  p = 0.023 |
| ACE count (per point) | 1.06 (0.98–1.15)  p = 0.137 | 1.08 (1.00–1.16)  p = 0.063 | 1.09 (1.00–1.17)  p = 0.043 |
| DASS-21 subscale (per point)ᵃ | 1.01 (0.99–1.03)  p = 0.485 | 1.00 (0.97–1.02)  p = 0.774 | 0.99 (0.96–1.02)  p = 0.401 |
| Low income (< $25,000) | 0.93 (0.50–1.72)  p = 0.811 | 0.95 (0.54–1.68)  p = 0.872 | 0.94 (0.53–1.67)  p = 0.842 |
| Age (per year) | 0.98 (0.96–1.00)  p = 0.043 | 0.98 (0.95–1.00)  p = 0.032 | 0.97 (0.95–1.00)  p = 0.025 |

PR, prevalence ratio; CI, confidence interval; MOS, Medical Outcomes Study; DASS-21, Depression Anxiety Stress Scales-21; ACE, adverse childhood experiences. All models are modified Poisson regressions with robust variance. Reference category for income: ≥ $25,000.

ᵃ Depression, anxiety, and stress respectively. The three subscales were entered in parallel models rather than simultaneously because they are strongly intercorrelated.

**Table S5.** Dietary quality (Mini-EAT score) as the outcome: parsimonious psychosocial model (robust linear regression; n = 127).

| **Predictor** | **β (95% CI)** | **p** |
| --- | --- | --- |
| Food insecure | −2.54 (−5.02 to −0.06) | 0.047 |
| Farmworker household | 2.54 (−0.34 to 5.41) | 0.086 |
| Education: high school / GED (vs some college+) | 2.07 (−0.53 to 4.67) | 0.121 |
| Education: less than high school (vs some college+) | 1.14 (−3.37 to 5.64) | 0.622 |
| Social support (MOS) score (per point) | 0.03 (−0.02 to 0.08) | 0.236 |
| ACE count (per point) | 0.08 (−0.41 to 0.57) | 0.750 |
| DASS-21 depression (per point) | −0.03 (−0.18 to 0.11) | 0.642 |
| Age (per year) | 0.07 (−0.05 to 0.19) | 0.249 |

*β, mean difference in Mini-EAT score (0–90 scale); CI, confidence interval; MOS, Medical Outcomes Study; DASS-21, Depression Anxiety Stress Scales-21; ACE, adverse childhood experiences. A higher Mini-EAT score indicates a healthier diet. This model reflects the same cross-sectional association reported in Table 3 under a different covariate set and does not provide independent confirmation of it; the two estimates should not be interpreted as separate findings. Reference category for education: some college or more.*

**Table S6.** Characteristics of participants included in (n = 124) versus excluded from (n = 75) the final parsimonious food-insecurity model.

| **Characteristic** | **Included (n = 124)** | **Excluded (n = 75)** | **SMD†** | **P value†** |
| --- | --- | --- | --- | --- |
| Food insecure (Hunger Vital Sign), n/N (%)‡ | 48/124 (38.7) | 33/63 (52.4) | −0.28 | 0.087 |
| Age, years, median (IQR) | 33.0 (26.0–41.2) | 32.5 (27.2–49.8) | −0.13 | 0.572 |
| Education, n/N (%) |  |  |  | 0.678§ |
| Some college or more | 75/124 (60.5) | 44/75 (58.7) | 0.04 |  |
| High school / GED | 37/124 (29.8) | 22/75 (29.3) | 0.01 |  |
| Less than high school | 12/124 (9.7) | 9/75 (12.0) | −0.08 |  |
| Low income (<$25,000), n/N (%) | 15/113 (13.3) | 15/67 (22.4) | −0.24 | 0.147 |
| Farmworker household, n/N (%) | 25/124 (20.2) | 22/75 (29.3) | −0.21 | 0.169 |
| Survey completed in Spanish, n/N (%) | 7/124 (5.6) | 13/75 (17.3) | −0.37 | 0.013 |
| Immigrant, n/N (%) | 32/124 (25.8) | 25/72 (34.7) | −0.20 | 0.196 |
| Discrimination total score, median (IQR) | 21.0 (16.0–27.0) | 21.0 (15.5–29.0) | −0.05 | 0.751 |
| MOS social support score, median (IQR) | 76.3 (52.6–94.7) | 75.0 (46.1–96.7) | 0.05 | 0.811 |
| ACE count, median (IQR)‖ | 2.0 (1.0–5.0) | 0.5 (0.0–4.5) | 0.36 | 0.131 |
| DASS-21 depression score, median (IQR) | 8.0 (2.0–14.0) | 9.0 (2.0–17.0) | −0.15 | 0.572 |
| DASS-21 anxiety score, median (IQR) | 8.0 (4.0–14.0) | 8.0 (2.0–17.0) | −0.07 | 0.983 |
| DASS-21 stress score, median (IQR) | 14.0 (7.0–18.0) | 14.0 (5.0–23.0) | −0.13 | 0.534 |
| Physically inactive (GPAQ), n/N (%) | 50/116 (43.1) | 18/58 (31.0) | 0.25 | 0.140 |
| BMI, kg/m², median (IQR) | 29.1 (26.4–34.2) | 28.0 (24.0–33.9) | 0.17 | 0.363 |
| Self-reported clinician-diagnosed diabetes, n/N (%) | 12/124 (9.7) | 7/53 (13.2) | −0.11 | 0.596 |
| Bang prediabetes score, median (IQR) | 3.0 (2.0–4.0) | 3.0 (2.0–5.0) | 0.08 | 0.729 |
| High Bang risk (score ≥ 5), n/N (%) | 24/108 (22.2) | 10/36 (27.8) | −0.13 | 0.503 |
| Mini-EAT diet score, median (IQR) | 60.0 (55.5–64.0) | 61.0 (55.8–67.0) | −0.09 | 0.502 |
| Poor diet quality (Mini-EAT), n/N (%) | 58/123 (47.2) | 29/68 (42.6) | 0.09 | 0.649 |
| SOIS acculturative stress, median (IQR)¶ | 1.6 (1.4–2.8) | 1.9 (1.4–2.6) | 0.08 | 0.889 |

*Abbreviations: ACE, adverse childhood experiences; DASS-21, Depression Anxiety Stress Scales-21; GPAQ, Global Physical Activity Questionnaire; IQR, interquartile range; MOS, Medical Outcomes Study; SMD, standardized mean difference; SOIS, Stress of Immigration Survey (mean score, range 1–5; Sternberg et al. 2016).*

*"Included" = complete data on all eight variables of the final parsimonious model (outcome, farmworker household, education, discrimination total score, MOS social support, ACE count, DASS depression, age); "excluded" = missing at least one. Denominators vary because of item-level missingness within each group; n/N shows the non-missing denominator.*

*† Standardized mean differences are the primary comparison (independent of sample size): for continuous variables, difference in means divided by the pooled standard deviation; for binary variables, difference in proportions on the same scale. P values are secondary: exact Wilcoxon rank-sum (continuous), Fisher's exact (binary), exact linear-by-linear trend test (education, ordered).*

*‡ Outcome observed for 63 of the 75 excluded participants; the remaining 12 are missing the Hunger Vital Sign items.*

*§ Trend test across the three ordered education levels (single test; per-level SMDs shown).*

*‖ ACE count is observed for only 14 of the 75 excluded participants (ACE missingness is the largest single cause of exclusion), so this comparison rests on a small, self-selected subgroup.*

*¶ Immigrant participants only (SOIS administered to immigrants by design): included n = 32, excluded n = 23.*

**Table S7.** Robust Wald versus nonparametric-bootstrap percentile confidence intervals for the final parsimonious food-insecurity model (n = 124, 48 food-insecure participants).

| **Predictor** | **PR** | **Robust 95% CI** | **Bootstrap percentile 95% CI** |
| --- | --- | --- | --- |
| Farmworker household | 1.65 | 1.07–2.55 | 1.01–2.75 |
| Education: high school / GED (vs some college+) | 1.29 | 0.82–2.04 | 0.78–2.18 |
| Education: less than high school (vs some college+) | 1.87 | 1.10–3.19 | 0.95–3.50 |
| Everyday Discrimination score (per point) | 1.00 | 0.97–1.03 | 0.97–1.03 |
| Social support, MOS score (per point) | 0.99 | 0.98–1.00 | 0.98–1.00 |
| ACE count (per point) | 1.05 | 0.97–1.14 | 0.96–1.16 |
| DASS-21 depression (per point) | 1.02 | 1.00–1.04 | 0.99–1.04 |
| Age (per year) | 0.98 | 0.97–1.00 | 0.96–1.00 |

*PR, prevalence ratio; CI, confidence interval; MOS, Medical Outcomes Study; DASS-21, Depression Anxiety Stress Scales-21; ACE, adverse childhood experiences.*

*Modified Poisson regression with robust (HC0 sandwich) variance; final parsimonious model as reported in Table 2 (n = 124, 48 food-insecure participants; 8 estimated parameters excluding the intercept, 6.0 events per parameter). Bootstrap: 5000 nonparametric resamples of the 124 complete cases with replacement, refitting the identical model in each; all 5000 resamples converged. Percentile intervals are the 2.5th–97.5th percentiles of the resampled prevalence ratios. Bootstrap and robust intervals were similar for all predictors except less-than-high-school education, whose bootstrap interval widens to include 1; this reflects the small size of that stratum (12 of 124 participants) and indicates that the education estimate should be interpreted with emphasis on its imprecision. The farmworker association is unchanged under the bootstrap.*
